# A Composite Porous Membrane Based on Derived Cellulose for Transient Gel Electrolyte in Transient Lithium-Ion Batteries

**DOI:** 10.3390/ma15041584

**Published:** 2022-02-20

**Authors:** Yuanfen Chen, Lanbin Zhang, Lin Lin, Hui You

**Affiliations:** School of Mechanical Engineering, Guangxi University, Nanning 530004, China; yuanfenchen@gxu.edu.cn (Y.C.); zhanglanbin@st.gxu.edu.cn (L.Z.)

**Keywords:** transient lithium-ion battery, transient gel electrolyte, cellulose membrane, cellulose gel electrolyte, transient electronics

## Abstract

The transient lithium-ion battery is a potential candidate as an integrated energy storage unit in transient electronics. In this study, a mechanically robust, transient, and high-performance composite porous membrane for a transient gel electrolyte in transient lithium-ion batteries is studied and reported. By introducing a unique and controllable circular skeleton of methylcellulose to the carboxymethyl cellulose-based membrane, the elastic modulus and tensile strength of the composite porous membrane (CPM) are greatly improved, while maintaining its micropores structure and fast transiency. Results show that CPM with 5% methylcellulose has the best overall performance. The elastic modulus, tensile strength, porosity, and contact angle of the optimized CPM are 335.18 MPa, 9.73 MPa, 62.26%, and 21.22°, respectively. The water-triggered transient time for CPM is less than 20 min. The ionic conductivity and bulk resistance of the CPM gel electrolyte are 0.54 mS cm^−1^ and 4.45 Ω, respectively. The obtained results suggest that this transient high-performance CPM has great potential applications as a transient power source in transient electronics.

## 1. Introduction

Transient electronics is an emerging technology that disintegrates or dissolves in its surrounding environment in a controlled manner once triggered. It has been attracting great attention in the fields of environmental monitoring, security, and biomedical applications [1,2,3,4]. A variety of transient electronic devices have been developed [5,6,7], but the lack of suitable power supply units has limited the application of transient electronics [8]. The requirement of disintegration or dissolution under a triggered signal is a great challenge in manufacturing transient energy storage units. Bettinger, Rogers, and Yin developed transient primary battery cells using biocompatible organic materials and metals [9,10,11]. These transient batteries could be applied in the field of transient biomedical electronics. However, the output voltage and energy density of these batteries are generally lower than traditional batteries and could not meet the energy requirement of most electronics. With the merits of high energy density and high output voltage [12], the transient lithium-ion battery is a promising power supply candidate for transient electronic devices, especially in applications such as harsh-environment monitoring, hardware security, and military applications [13,14]. Kun and his colleagues reported transient lithium-ion batteries triggered by cascade reactions [13,15]. V_2_O_5_ was used as a cathode, lithium metal and its alloy were used as an anode, and polyvinylpyrrolidone and polyethylene oxide were used as an electrolyte separator. Montazami and his group reported a transient lithium-ion battery triggered by the combination of chemical dissolution and physical disintegration [16]. The cathode and anode were LiCoO_2_ and Li_4_Ti_5_O_12_, respectively, and water-soluble paper was used as an electrolyte separator. The transiency of the lithium-ion battery mainly depends on the transient property of the encapsulation material, electrode binder material, and electrolyte separator/membrane. Among the three components, the transient electrolyte separator/membrane is the most challenging, for its electrochemical property, mechanical property, and morphology requirements besides the triggered transiency. High-performance transient electrolytes are in demand [17,18].

Gel electrolytes are widely studied because of their advantages in safety, ionic conductivity, and flexibility [19,20,21,22,23]. Nowadays, most high-performance gel electrolytes are produced by absorbing liquid electrolytes into porous membranes [24]. To obtain a transient gel electrolyte, the porous membrane must be triggered transiently, as they are especially favorable to degradation. Cellulose and its derivatives meet this requirement [20,25] and have been increasingly used as the membrane for gel electrolytes in lithium-ion batteries [24,26,27,28], but the transient property has not been considered in previous studies. The degradation time for cellulose in the natural environment varies from months to years, while some cellulose derivatives can dissolve in water in several minutes. Transient gel electrolyte membranes made from water-soluble cellulose derivatives are favorable for applications that require rapid transiency, such as hardware security and military. Zhu et al. reported a porous carboxymethyl cellulose membrane, produced by adding N, N-dimethylformamide (DMF), and then evaporated the agent via heating at 80 °C. The membrane could be used as a gel polymer electrolyte for lithium-ion batteries, but the tensile strength of the membrane with optimized porosity was relatively low [29]. The weak mechanical property is a general disadvantage for water-soluble porous cellulose membranes. Some chemical modifications, such as cross-linking, could improve the mechanical strength of the cellulose porous membrane [20], but would impair its water solubility. Therefore, it is necessary to seek methods that could improve the mechanical properties of porous cellulose membranes while also maintaining their rapid transiency. 

In this paper, a composite porous membrane based on derived cellulose for a transient gel electrolyte in transient lithium-ion batteries was reported. The mechanical property of the carboxymethyl cellulose membrane was greatly improved by adding methylcellulose; meanwhile, the micropore structure and rapid transiency property of the membrane were maintained. The porosity, liquid electrolyte uptake ratio, wettability, transiency, and ionic conductivity of different carboxymethyl cellulose and methylcellulose ratios were characterized to optimize the CPM composition. The potential application of the CPM as a transient gel electrolyte membrane was demonstrated by its electrochemical performance.

## 2. Materials and Methods

### 2.1. Fabrication of Composite Porous Membrane

The CPM was fabricated by the solution-casting method. Firstly, a certain amount of carboxymethyl cellulose (CMC) (viscosity: 600–3000 mPa·s, Macklin, Shanghai, China) was added to 30 mL of distilled water and stirred at 65 °C for 1 h. Then, a certain amount of methylcellulose (MC) (viscosity: 400 mPa·s, Macklin) was added to the clear CMC solution and stirred at 45 °C for 1 h. Subsequently, 1 mL of N, N-dimethylformamide (DMF) (AR, Fuyu Chemical, Tianjin, China) was added to the clear CMC-MC solution and stirred at 45 °C for 0.5 h. The clear solution was then cast into a polytetrafluoroethylene mold and dried at 80 °C for 4 h to obtain CPMs. The total amount of CMC and MC was 0.2 g, with the MC ratio ranging from 0–10 wt%, as shown in Table 1. The thickness of CPMs was approximately 50 μm.

### 2.2. Porosity Measurement and Liquid Electrolyte Uptake Ratio measurement

The porosity of CPM was calculated according to Equation (1).

(1) $$P=\frac{m_{e}-m_{i}}{\rho V}\times 100\%$$

where *m_i_* and *m_e_* are the weights of the CPM before and after soaking in n-Butanol (AR, ChronChem), respectively. *ρ* is the density of n-Butanol, and *V* is the volume of the CPM sample.

The CPM was immersed in the liquid electrolyte for 12 h. The uptake of the liquid electrolyte ratio (*η*) can be calculated by Equation (2)

(2) $$\eta =\frac{W_{1}-W_{0}}{W_{0}}\times 100\%$$

where *W*_0_ and *W*_1_ are the weights of the CPM before and after the absorption of the liquid electrolyte, respectively.

### 2.3. Microscopic Morphology Characterization 

The microscopic morphology of the CPM was measured by a scanning electron microscope (SEM, ZEISS, Sigma 500, Jena, Germany). The voltage was set as 5 kV. Since the CPM itself was not conductive, it was sprayed with gold for 30 s in a gold-spraying instrument (Gressington, 108Auto, Redding, CA, USA) before observation.

### 2.4. Mechanical Property Characterization

The stress–strain curves of CPMs and their elastic modulus were tested using a tensile testing machine (SUNS, UTM2502, Shenzhen, China) with a stretching speed of 1 mm min-1. The sample size was 16 mm × 10 mm. Six samples of each type were tested.

### 2.5. Transiency Characterization

The CPM was placed in a beaker, followed by the addition of 30 mL of distilled water. The dissolution time was recorded, and a dissolution picture was taken every 5 min until the CPM was completely dissolved. Following this, the samples were taken out of the water, dried in the oven, and the remaining weight was weighed, after soaking in water for 5, 10, 15, and 20 min. Three samples were measured for each time period.

### 2.6. Contact Angle Measurement

The contact angle of the CPM was measured using a contact-angle measuring device (SINDIN, SDC-200S, Dongguan, China). Six samples of each type were tested. The contact angle was estimated right after the liquid electrolyte was dropped on the sample surface to avoid liquid electrolyte absorption.

### 2.7. Ionic Conductivity Measurement

The electrochemical impedance spectra (EIS) of the gel polymer electrolytes were tested using an electrochemical workstation (AMETEK, PARSTAT 4000A, Berwyn, PA, USA) in the frequency range of 100 Hz–100 kHz. The gel polymer electrolyte was obtained by immersing the CPM in a liquid electrolyte of 1 M LiPF_6_ in ethylene carbonate/dimethyl carbonate/ethyl methyl carbonate (1/1/1, *v*/*v*/*v*, DoDoChem, Suzhou, China) for 12 h in a glove box. The size of the CPM was 15 mm × 15 mm. The gel polymer electrolyte was sandwiched between two square, stainless-steel electrodes (304, Shanghai, China) with an area of 1.96 cm^2^ and tested in a temperature range of 25 °C–75 °C. The ionic conductivity was calculated by Equation (3) [30].

(3) $$\sigma =\frac{t}{R_{b}A}$$

where *t* is the thickness of each sample, *R_b_* is the bulk resistance, and *A* is the surface area.

### 2.8. Full Cell Performance

The gel polymer electrolyte was sandwiched between the anode and cathode to assemble the battery cell. Copper foil coated with graphite (Canrd, Dongguan, China) was used as the anode and aluminum foil coated with LiFePO_4_ (Canrd, Dongguan, China) was used as the cathode. The size of the anode and cathode was 14 cm × 14 cm, and the area of the gel polymer electrolyte was slightly larger than that of electrodes to avoid a short circuit. The EIS of the full cell was tested using an electrochemical workstation (AMETEK, PARSTAT 4000A, Berwyn, PA, USA).

## 3. Results and Discussion

### 3.1. Porous CPM 

**Figure 1:**
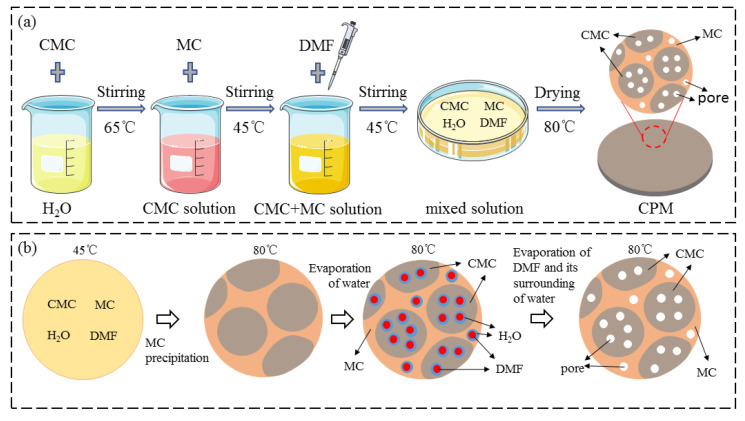
(**a**) Schematic illustration of the process for the preparation of CPM; (**b**) schematic illustration of nanoscale pore formation in CPM.

**Figure A1:**
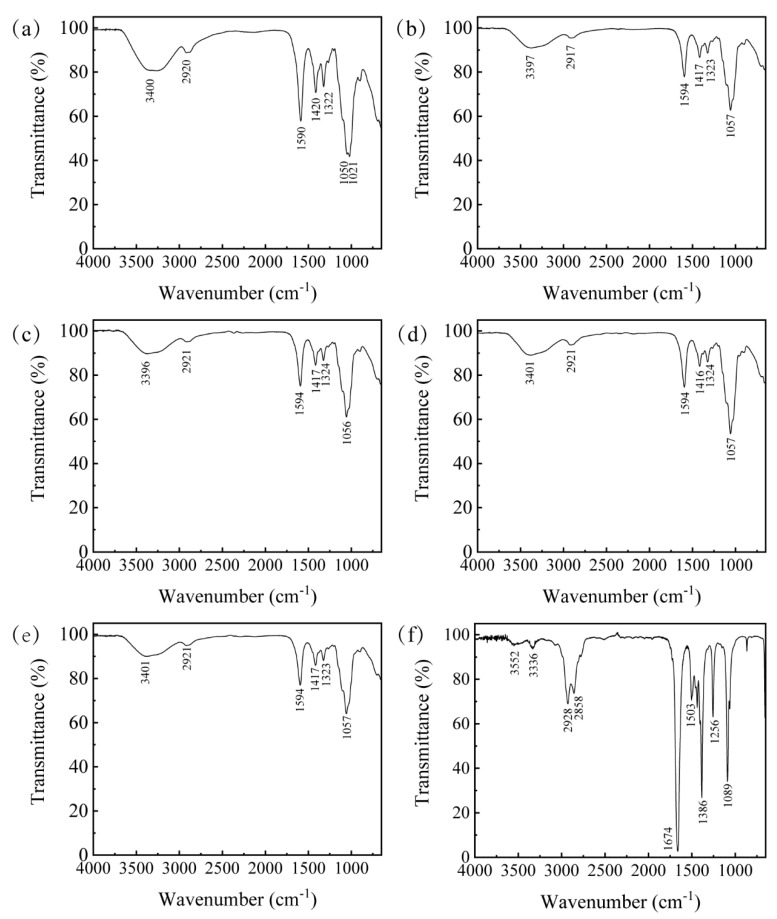
FTIR spectra: (**a**) CPM-0; (**b**) CPM-3; (**c**) CPM-5; (**d**) CPM-7; (**e**) CPM-10; (**f**) DMF.

The preparation process of the CPM was shown in Figure 1. The CMC, MC, and DMF were dissolved in water sequentially and then cast in a mold to make the CPM (Figure 1a). A non-solvent evaporation method was used to form nanoscale pores in the CPM, as shown in Figure 1b. The boiling point of the non-solvent DMF (153 °C) is higher than the water solvent (100 °C), so the evaporation rate of water is much faster at 80 °C. After the water evaporated, the CPM became a solid-phase matrix containing fine droplets of DMF. Finally, DMF evaporated, and the CPM matrix remained unchanged, forming a nanoscale porous structure. The complete evaporation of DMF is validated by the FTIR spectrum in Appendix A, Figure A1. 

**Figure A2:**
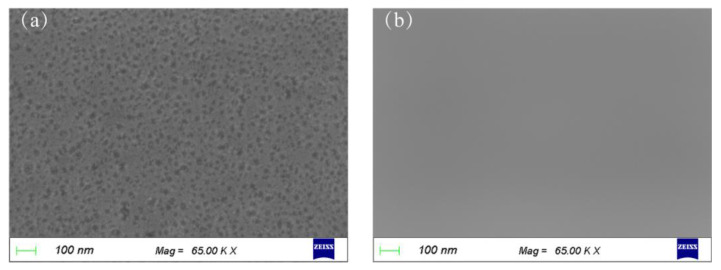
SEM micrograph of: (**a**) Pure CMC membrane; (**b**) pure MC membrane.

**Figure A3:**
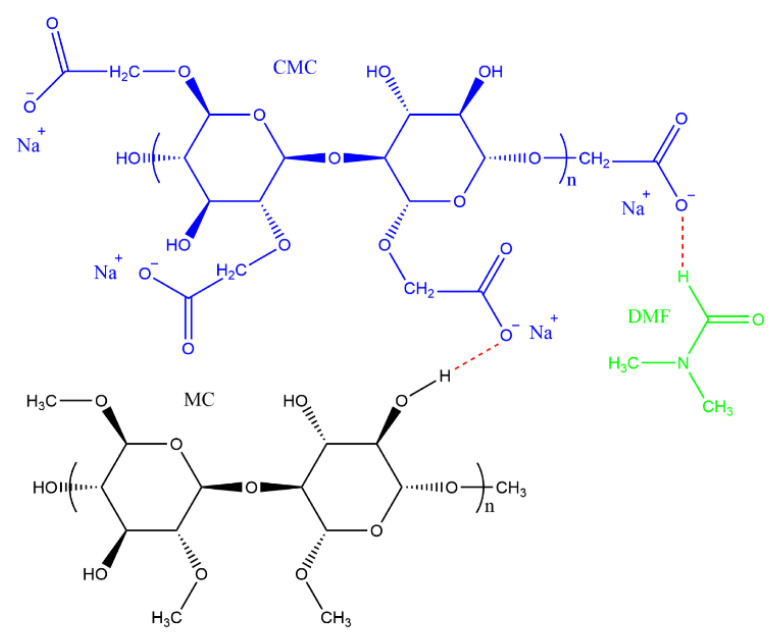
Possible interaction between CMC, DMF, and MC.

Particularly, the non-solvent evaporation method worked for the pure CMC membrane, but not for the pure MC membrane, as shown in Appendix A, Figure A2. That might be because DMF has a stronger interaction with the O-Na bond in CMC, and less interaction with MC. The possible chain interaction between CMC, DMF, and MC is illustrated in Appendix A, Figure A3. It was inferred that the DMF drop mainly existed in the CMC-dominated phase in the CPM, as did the micropores. Since the mechanical property of MC is higher than that of CMC, adding MC would improve the mechanical property of the CPM, but may also impair the micropore structure of the CPM, thus the MC content should be carefully controlled. The effect of the MC content on the mechanical property and micropore structure will be studied in the following sections.

### 3.2. Morphology

**Figure 2:**
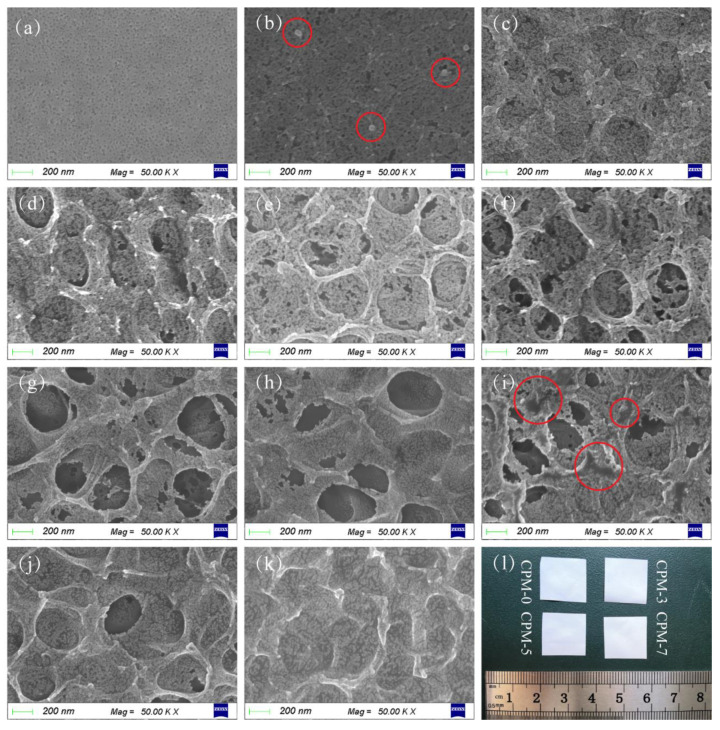
SEM micrographs of CPMs for surface in contact with air: (**a**–**k**) correspond to (CPM-0~CPM-10), respectively; red circles in (**b**), dot-like structure; red circle in (**i**), dense non-porous structure; (**l**) photographs of CPM-0, CPM-3, CPM-5, and CPM-7.

The microscopic morphology of the CPM observed by SEM is shown in Figure 2. It can be clearly seen that there was a large number of nanoscale pores inside the CPMs, which agreed with the previous report that DMF evaporation would introduce pores in CMC [29]. The porous structure is essential for forming the gel electrolyte as micropores enable high liquid electrolyte uptake, thus providing migration channels for lithium ions.

The porous structure of the CPM changed with different ratios of CMC and MC. It could be seen that the pure CMC membrane (Figure 2a) had a uniform nanoscale porous structure (about 20 nm). With a 1% addition of MC (Figure 2b), dot-like structures started to appear on the surface of the composite membrane (marked by the red circles). With the 2–3% addition of MC content, the surface of the membrane started to form faintly visible circular structures (Figure 2c,d), which was thought to be an MC-dominated structure. With the further increase in MC content, the surface of the membrane started to show a complete monolayer circular skeleton structure (Figure 2e,f, 4–5% of MC), while the nanoscale porous structure in the CMC-dominated phase below the circular skeleton structure was still clearly visible. Moreover, nanoscale pores were also present on the circular skeleton structure. As the MC content continued to increase, the monolayer circular skeleton structure formed by the MC-dominated phase developed into a multilayer structure (Figure 2g,h, 6–7% of MC). The nanoscale porous structure still existed on the skeleton structure at this MC ratio. As the MC content increased to 8%, although the CPM still showed a multilayer skeleton structure, the multilayer circular skeleton started to form a dense non-porous structure (Figure 2i, marked by red circles). The multilayer circular skeleton structure disappeared when the MC content increased to 9–10% (Figure 2j,k). Unlike the microstructure, the macro-appearances of different types of CPM (Figure 2l) were not significantly different.

As can be seen from Figure 2a–k, the dimension of the micropores changed with MC content. The dimension of the micropores increased with the MC content from 0–5 wt% (Figure 2a–f). As the MC content continued to increase, the circular skeleton structure formed by the MC-dominated phase became more compact (Figure 2g–k). It was expected that the free volume in the CPM would first increase with MC content, then decrease.

**Figure 3:**
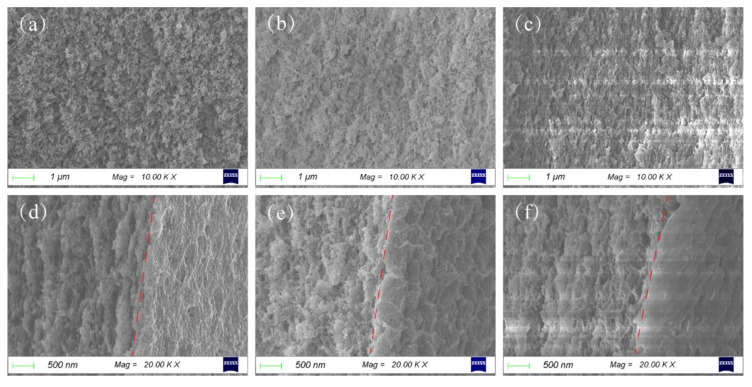
SEM micrographs of CPMs: (**a**–**c**) cross-section micrographs of CPM0, CPM5, and CPM10, respectively; (**d**–**f**) micrographs of the junction between the cross-section (left side of the red dashed line) and the surface (right side of the red dashed line) of CPM0, CPM5, and CPM10, respectively.

**Figure A4:**
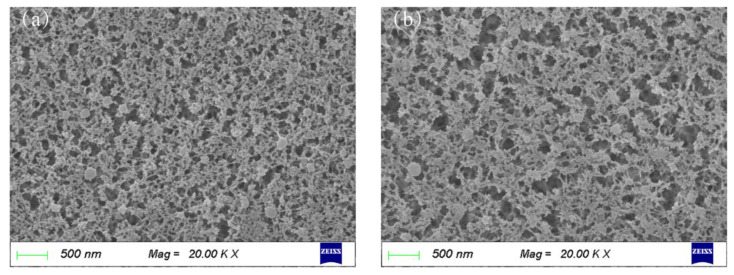
SEM micrograph of the bottom surface of CPMs: (**a**) CPM-0; (**b**) CPM-5.

The microstructure of the bottom surface was almost identical for CPMs with different MC content, as shown in Appendix A, Figure A4. The microscopic morphology of the CPM cross-section observed by SEM is shown in Figure 3. The morphologies of the CMC-dominated phase for different types of CPMs were almost identical (Figure 3a–c). The morphology was homogenous for the entire cross-section of CPM-0 (Figure 3d). A more-condensed layer near the surface could be seen for CPM-10 (Figure 3f), corresponding to Figure 2k. For CPM-5, the layer structure was faintly visible. The nonuniformity of the CPM in the thickness direction might be caused by the gelation of MC at the early stage of the water evaporation process. MC dissolved in water and formed a clear solution at a temperature lower than 50 °C, and started to turn to gel when the temperature was higher [31]. When the solution was dried at 80 °C, MC turned into a gel first, then water evaporated, and finally, the CPM became a solid-phase matrix with the MC-dominated phase mainly exiting through the upper surface of the CPM.

Based on the SEM micrograph analysis, five CPMs, CPM-0, CPM-3, CPM-5, CPM-7, and CPM-10, which showed relatively obvious changes in their morphology, were selected to continue the following study.

### 3.3. Mechanical Properties

**Figure 4:**
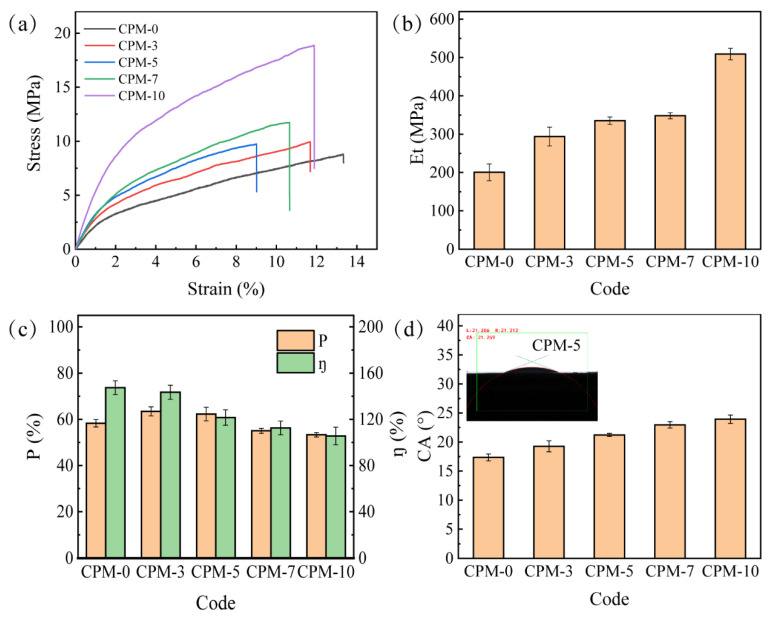
(**a**) The strain–stress curves of CPMs; (**b**) elastic modulus of CPMs; (**c**) porosity and liquid electrolyte uptake ratio of CPMs; (**d**) contact angle between liquid electrolyte and CPMs.

The mechanical property of the membrane is very important for its practical application. Membranes with high mechanical properties can withstand higher external forces and maintain the porous structure to ensure the migration channels for lithium ions. Figure 4a shows the tensile stress–strain curves of CPMs with different CMC and MC contents. The pure CMC membrane (CPM-0) had weak mechanical properties. The mechanical properties of the CPM improved as the MC content increased. It was assumed that the circular skeleton structure on the surface of CPM contributed to the mechanical property improvement. With the increase in MC content from 0% (CPM-0) to 10% (CPM-10), the tensile strength of the CPM increased from 8.81 MPa to 18.88 MPa, increasing by 114.30%. As shown in Figure 4b, the elastic modulus of CPM increased from 200.69 MPa (CPM-0) to 508.99 MPa (CPM-10), increasing by 153.62%. Comparing the tensile strength increment trend with the morphology change trend, the unique circular skeleton structure formed by the MC-dominated phase was the main reason for the mechanical property improvement.

### 3.4. Porosity and Liquid Electrolyte Uptake Ratio

The porous membrane needs to absorb and hold enough liquid electrolytes to ensure lithium-ion migration, so the porosity and liquid electrolyte uptake ratio (*η*) of the CPMs were analyzed. The porosity of CPMs calculated according to Equation (1) is shown in Figure 4c (brown color). It could be seen that the porosity of the CPM increased with an increasing MC content from 0–3 wt%, levelized, and then decreased with an increasing MC content from 5–10 wt%. The minimum porosity was 53.34% with 10 wt% MC. The porosity–MC content correlation agreed with the microstructure change trend in Figure 2. Generally, the porosity of the commercial membrane is 30–70%, and a porosity higher than 40% is favorable [32,33]. The porosity of all the CPMs meet this requirement. The liquid electrolyte uptake ratio of CPMs calculated according to Equation (2) is shown in Figure 4c (green color). Unlike the porosity change trend, the liquid electrolyte uptake of the CPM decreased from 147.36% (CPM-0) to 105.56% (CPM-10). The possible reason might be that MC has less interaction with the electrolyte than CMC. 

### 3.5. Wettability and Contact Angle

The wettability of the CPM affects its ability to absorb and hold the liquid electrolyte. Here, the contact angle (CA) was measured to evaluate the wettability of CPMs [32]. As shown in Figure 4d, the contact angles of CPM-0, CPM-3, CPM-5, CPM-7, and CPM-10 were 17.36°, 19.27°, 21.22°, 22.96°, and 23.92°, respectively. The contact angle of the CPM increased slightly as the MC content increased, but all of them were much less than 90°, which indicated good wettability of all the CPMs.

### 3.6. Transiency

**Figure 5:**
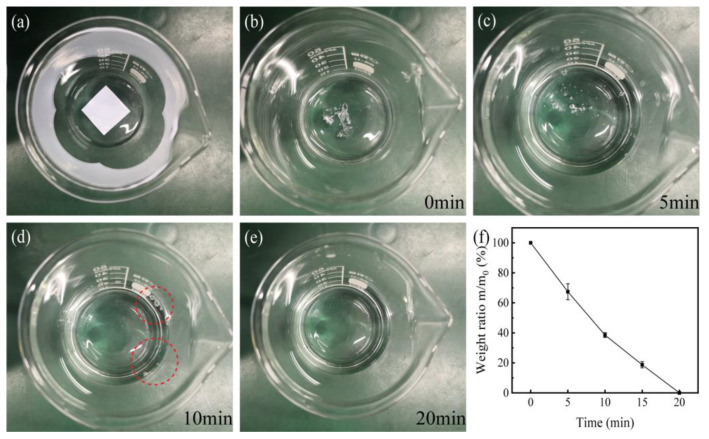
The triggered transiency of CPM: (**a**) Initial state without adding water; (**b**) the moment water was added; (**c**) dissolution status after 5 min; (**d**) dissolution status after 10 min, the bubbles left were marked by red circles; (**e**) dissolution status after 20 min; (**f**) the weight ratio as a function of dissolution time.

Fast and controlled transiency is required for transient battery applications. For degradable cellulose, the triggered transiency would be dissolved in water or degraded in soil. Here the dissolution ability of the CPM was studied. Figure 5 shows the dissolution process of CPM-5 in DI water. CPM-5 was rapidly infiltrated into a transparent gel when DI water was added (Figure 5b). Five minutes later, CPM-5 was partially dissolved, and bubbles could be observed in the CPM-5 gel (Figure 5c). After 10 min, the CPM-5 gel was dissolved and most of the bubbles disappeared (Figure 5d, the bubbles left were marked by red circles). After 20 min, the CPM-5 gel was completely dissolved, and the bubbles completely disappeared (Figure 5e). That is, the water-triggered transient time for CPM-5 was less than 20 min. All types of CPMs showed almost the same dissolution process, indicating that MC content has little effect on the transiency of CPMs. The weight ratio as a function of the dissolution time is shown in Figure 5f. The remaining weight after 0, 5, 10, 15, and 20 min of dissolution time was 100%, 67.42%, 38.53%, 18.74%, and 0% of the original weight, respectively.

### 3.7. Ionic Conductivity

**Figure 6:**
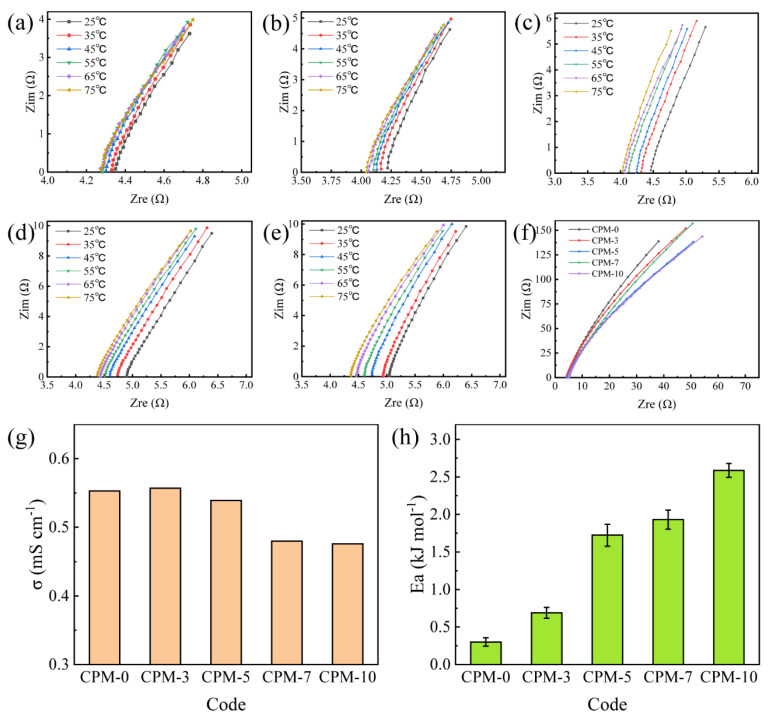
EIS plots at different temperatures for the CPM gel electrolyte: (**a**) CPM-0; (**b**) CPM-3; (**c**) CPM-5; (**d**) CPM-7; (**e**) CPM-10; (**f**) EIS plots of the samples at 25 °C at a frequency of 100 Hz–100 kHz; (**g**) ionic conductivity of CPM gel; (**h**) activation energy of CPM gel.

Impedance is one of the most important properties of gel polymer electrolytes and characterizes the migration ability of lithium ions. The electrochemical impedance spectra (EIS) of the activated CPM gel electrolyte with different MC contents are shown in Figure 6a–f. As can be seen, the EIS of all samples was approximately linear, indicating that all CPM gel electrolyte samples had approximately pure resistive behavior at a high frequency. The EIS of the CPM gel polymer electrolyte in the temperature interval of 25 °C to 75 °C is shown in Figure 6a–e. Figure 6f shows the EIS plots of the samples at 25 °C for a frequency of 100 Hz–100 kHz. It can be seen that upon keeping the MC content constant, the bulk resistance (*R_b_*) decreased as temperature increased; upon keeping the temperature constant, *R_b_* increased as the MC content increased. 

The ionic conductivity was calculated according to Equation (3). At 25 °C, the ionic conductivity of CPM-0, CPM-3, CPM-5, CPM-7, and CPM-10 were 0.55, 0.56, 0.54, 0.48, and 0.47 mS cm^−1^, respectively. It could be seen that the ionic conductivity of the CPM increased with an increasing MC content from 0–3 wt%, and then decreased with an increasing MC content from 5–10 wt%. The ionic conductivity–MC content correlation agreed with the microstructure change trend and the porosity–MC content correlation.

It is clear from Figure 6a–e that the bulk resistance of the gel polymer electrolyte decreased with increasing temperature, and therefore the ionic conductivity must consequently keep increasing, showing typical Arrhenius behavior [20]. The activation energy (*Ea*) for the movement of lithium ions could be calculated according to Equation (4):

(4) $$\sigma =A\exp\left(-\frac{Ea}{R_{t}T}\right)$$

where *σ* is the ionic conductivity of the CPM gel polymer electrolyte, *R_t_* is the thermodynamic gas constant, *Ea* is the activation energy, *A* is the pre-exponential factor, and *T* is the temperature.

As shown in Figure 6h, the activation energies for CPM-0, CPM-3, CPM-5, CPM-7, and CPM-10 were 0.30, 0.69, 1.72, 1.93, and 2.59 kJ mol^−1^, respectively. As the MC content increased, the *Ea* required for ion motion increased. This indicated that the movement of lithium ions becomes increasingly difficult as the MC content increases. It was assumed that the circular skeleton structure, especially the non-porous multi-layer structure, hindered the movement of lithium ions.

### 3.8. Battery Performance

**Figure 7:**
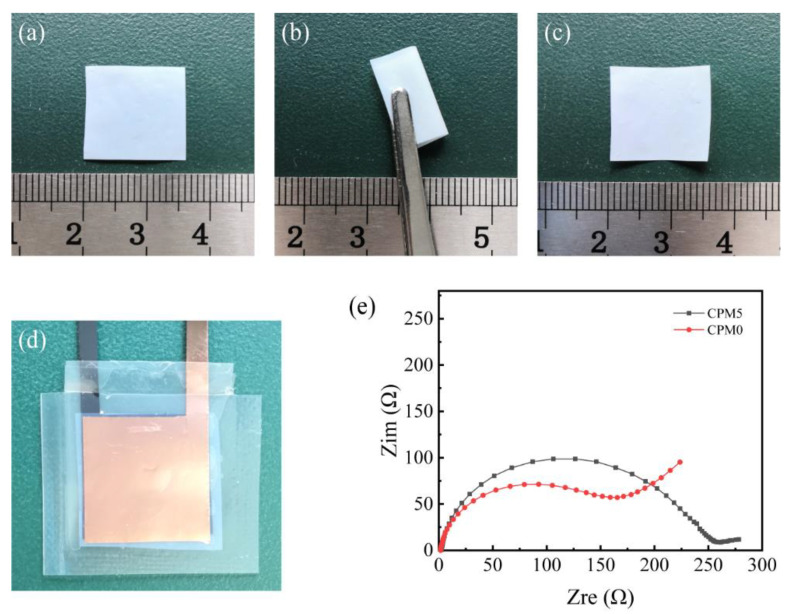
(**a**–**c**) Photographs of CPM-5 before folding, after folding, and released, respectively. (**d**) Photograph of a LiFePO_4_/CPM-5 gel electrolyte/graphite battery cell. (**e**) The EIS of the assembled LiFePO_4_/CPM gel electrolyte/graphite cell.

Considering both the mechanical and electrochemical properties of all samples, CPM-5 possessed the best overall performance, so CPM-5 was selected to continue the study. Figure 7a–c shows the appearance morphology of CPM-5 for the folding test. It can be seen that CPM-5 was flexible and could be folded and released without damage. Figure 7d shows a photograph of the LiFePO_4_/CPM-5 gel electrolyte/graphite battery cell.

The compatibility between the CPM gel electrolyte and electrodes is an important property for the interfacial stability of the battery cell. Figure 7e shows the EIS of the LiFePO_4_/CPM gel electrolyte/graphite battery cell. The EIS consisted of a high-frequency semicircular region and a low-frequency linear region, with the diameter of the semicircle corresponding to the charge transfer resistance (*R_ct_*), and the intercept of the *x*-axis being the bulk resistance (*R_b_*). For CPM-0, *R_b_* is calculated to be 4.34 Ω and *R_ct_* is calculated to be 200 Ω. For CPM-5, *R_b_* is calculated to be 4.45 Ω and *R_ct_* is calculated to be 260 Ω. The interface impedance for both CPM-0 and CPM-5 was low, and the addition of 5 wt% of MC did not significantly increase the impedance. 

## 4. Conclusions

The addition of MC caused the formation of a unique circular skeleton structure on the CMC porous membrane, which significantly improved the mechanical properties of the CPM, and while also maintaining its micropore structure and rapid transiency in water. Upon increasing the MC content from 0 wt% to 10 wt%, the elastic modulus of the CPM increased from 200.69 MPa to 508.99 MPa, increasing by 153.62% and the tensile strength of the CPM increased from 8.81 MPa to 18.88 MPa, increasing by 114.30%. The dimension of the micropores increased with an increasing MC content from 0–3 wt%, as did the porosity and ionic conductivity. As the MC content increased further, the circular skeleton structure formed by the MC-dominated phase became more compact, and both the porosity and ionic conductivity of the CPM decreased. The MC content had little effect on the transiency, and the water-triggered transient time was approximately 20 min. The ionic conductivity of the CPM slightly decreased from 0.55 mS cm^−1^ to 0.47 mS cm^−1^ at 25 °C, as the MC content increased from 0%wt to 10%wt. By optimizing the MC ratio, the obtained composite porous membrane with 5%wt MC (CPM-5) exhibited the best overall performance, with both high mechanical properties and ionic conductivity. For the LiFePO_4_/CPM-5 gel electrolyte/graphite battery cell, the bulk resistance and charge transfer resistance were 4.45 Ω and 260 Ω, respectively, at room temperature. The reported optimized CPM could be applied in transient lithium-ion batteries due to its high performance and intrinsic transient properties.

## Figures and Tables

**Table 1:** Code and composition of CPM.

Code	CPM-0	CPM-1	CPM-2	CPM-3	CPM-4	CPM-5	CPM-6	CPM-7	CPM-8	CPM-9	CPM-10
CMC	100%	99%	98%	97%	96%	95%	94%	93%	92%	91%	90%
MC	0%	1%	2%	3%	4%	5%	6%	7%	8%	9%	10%

## Data Availability

Not applicable.

## Appendix A

The characteristic absorption peak of CPM-0 (Figure A1a) is observed at 3400, 2920, 1590, and 1420 cm^−1^, which correspond to the stretching vibration of O-H, C-H(aliphatic), COO^−^(asymmetric), and COO^−^(symmetric), respectively. The characteristic absorption peak of MC was observed at 3000–2800, 1646, 1458, and 1300–1100 cm^−1^, which correspond to the stretching vibration of C-H, C–C ring stretching, C-H, and C-O. The characteristic absorption peaks of MC roughly overlapped with those of CMC, so the FTIR of CPM-3, CPM-5, CPM-7, and CPM-10 (Figure A1b–e) was similar to that of CPM-0 (Figure A1a). Besides, there was no characteristic absorption peak of DMF (Figure A1f, 3552, 3336, 1674, 1386), which indicated that there was no residual of the pore-forming agent DMF.

There was no significant difference in the microstructure of the bottom surface of the CPM with different MC contents, as shown in Figure A4a (CPM-0) and A4b (CPM-5).
